# Identification of a novel sequence type of *Escherichia coli* as the causative agent of pyelonephritis and bloodstream infection

**DOI:** 10.1099/jmmcr.0.005061

**Published:** 2016-10-31

**Authors:** Micheál Mac Aogáin, Helen Miajlovic, Geraldine Moloney, Sanjay H. Chotirmall, Thomas R. Rogers, Stephen G. J. Smith

**Affiliations:** 1 Department of Clinical Microbiology, Sir Patrick Dun Research Laboratory, School of Medicine, Trinity College, Dublin, Ireland; 2 Lee Kong Chian School of Medicine, Nanyang Technological University, Singapore

**Keywords:** Pyelonephritis, bacteraemia, *Escherichia coli* ST-458, Virulence factors, Antimicrobial resistance, Infected renal parenchymal cyst

## Abstract

**Introduction:**

Globally, extra-intestinal pathogenic *Escherichia coli* are one of the predominant causative agents of bacteraemia.

**Case presentation:**

This case report outlines a presentation of community-acquired pyelonephritis and secondary bloodstream infection in an 81-year-old man. Laboratory investigations revealed that the causative isolate was a multi-drug-resistant *E. coli* of a novel multi-locus sequence type. This sequence type (ST) was designated ST-458 and was most closely related to the globally prevalent ST-131 lineage.

**Conclusion:**

This is the first report of a novel *E. coli* ST, ST-458, which caused pyelonephritis and bacteraemia.

## Introduction

Extra-intestinal pathogenic *Escherichia coli* (ExPEC) are the leading cause of Gram-negative bloodstream infections (de Kraker *et al.*, 2013). Bacteraemia most frequently arises from an ascending infection originating in the urinary tract (Al-Hasan *et al.*, 2009; Jackson *et al.*, 2005). Therefore, uropathogenic *E. coli* (UPEC) are often the causative agents of bacteraemia and are well adapted to survival in the host environment. Globally, rates of *E. coli* bacteraemia are on the rise (de Kraker *et al.*, 2013). Antimicrobial resistance amongst *E. coli* has also been increasing and is of great concern to public health. Particularly worrying are the increasing numbers of *E. coli* producing extended spectrum β-lactamases (ESBL) and multi-drug-resistant (MDR) *E. coli* (Alhashash *et al.*, 2013; de Kraker *et al.*, 2013).

Currently, *E. coli* sequence type (ST) ST-131 is the pre dominant global lineage causing bloodstream infections (Nicolas-Chanoine *et al.*, 2014). Fluoroquinolone resistance and production of ESBL is common amongst strains of this ST. In this report, a novel ST, designated ST-458, was identified as the causative agent of *E. coli* pyelonephritis and bacteraemia in an 81-year-old man.

## Case report

An 81-year-old man presented to the emergency department with acute onset of fevers, rigors and vomiting. In the preceding week, he had a non-specific prodromal malaise. The patient’s past medical history was significant for diabetes mellitus, ischaemic heart disease, hypertension, benign prostatic hypertrophy, type 1 hypersensitivity reaction to penicillin and surgical resection of a colorectal carcinoma 5 years previously.

Significant findings of initial examination included fever and generalised abdominal tenderness. Initial laboratory findings were indicative of acute kidney injury, with urea measured at 15.8 mmol l^−1^, creatinine at 193 µmol l^−1^, bicarbonate at 17 mmol l^−1^ and eGFR at 30 ml min^−1^. The patient’s haematology profile revealed iron deficiency anaemia, with haemoglobin measurement of 7.3 g/dl and mean corpuscular volume of 79.2 fl. His total white cell count was 8.6×10^9^ l^−1^, with neutrophils of 8.0×10^9^ l^−1^ and lymphocyte count of 0.5×10^9^ l^−1^.

Blood cultures sent from the emergency department were flagged for growth at 12 h, with identification of *E. coli*. Antibiotic susceptibility testing showed resistance to ampicillin, amoxicillin, co-amoxiclav, cefuroxime, cefalotin, ciprofloxacin and gentamicin (Table S1, available in the online Supplementary Material). *E. coli*, with a corresponding antibiogram, was recovered from a mid-stream urinalysis.

The patient underwent a renal ultrasound, which revealed a hypoechoic cystic area, with dimensions of 7×3.2 cm. This was confirmed by a computed tomography on the kidney, which showed a cystic structure with an enhancing rim and associated perinephric stranding. There were no internal septations or nodules within the cyst.

The patient was diagnosed with community-acquired pyelonephritis, with infected cyst, and secondary bacteraemia due to *E. coli*. Taking into account the patient’s type 1 hypersensitivity to penicillin, initial empiric treatment in the emergency department consisted of ciprofloxacin and gentamicin. This was escalated to Meropenem in light of the associated antibiogram. Further testing did not find evidence of either ESBL or AmpC production and confirmed susceptibility to Aztreonam. This enabled de-escalation to IV Aztreonam.

Drainage of the cyst was performed under ultrasound and fluoroscopic guidance within the interventional radiology department. Fluid from this cyst had microscopic findings of many pus cells and moderate amounts of Gram-negative bacilli seen by Gram stain. This fluid culture was positive for *E. coli* with an identical antibiogram to the blood culture isolate.

## Investigations

### Whole genome sequencing

The *E. coli* isolate cultured from the patient’s bloodstream was named ECBSI31-SJH. Chromosomal DNA was extracted from ECBSI31-SJH and used to prepare a DNA sequencing library (2×250 bp PE) using the NexteraXT protocol (Illumina). The resultant library was sequenced on an Illumina MiSeq platform at the TrinSeq sequencing facility (Trinity College Dublin). Reads were assembled *de novo* using the Simplicity^TM^ pipeline (v1.2, NSilico Life Sciences) (Walsh *et al.*, 2013). A draft assembly has been deposited in GenBank (accession number LULC00000000).

### Phylogenetic analysis of ECBSI31-SJH

Multi-locus sequence typing (MLST) was performed *in silico *according to the Pasteur MLST *E. coli* typing scheme. The MLST analysis revealed that ECBSI31-SJH was of a novel ST designated ST-458 and was most closely related to ST-43. When compared to ST-43, a single allele, *trpA*, differs in ST-458 by a single mutation (A272T). Sequence type 43 is equivalent to ST-131 in the Achtman typing scheme. The alleles used in MLST analysis in the Achtman scheme differ from those used in the Pasteur MLST typing scheme. According to the Achtman scheme, ST-458 is also a novel ST and differs from ST-131 by a single mutation in the *mdh* locus (G72A). MLST data was deposited in the online repository of Institut Pasteur (http://bigsdb.web.pasteur.fr/). WGS data revealed ECBSI31-SJH to be closely related to other ST-131 strains within the described clade C (ST43/H30R) sub-lineage (Fig. S1).

### Virulence factors of ECBSI31-SJH

ECBSI31-SJH harbours virulence factors characteristic of ExPEC including multiple genes across a range of categories including adhesins, protectins, iron acquisition systems, toxins and other genes associated with virulence. These are listed in Table 1. All 131 UPEC-specific genes identified by Lloyd *et al.* (2007), were present in ECBSI31-SJH. Analysis of the *fimH* gene sequence confirmed the presence of the *fim*H30 allele (Weissman *et al.*, 2012).

**Fig 1.**
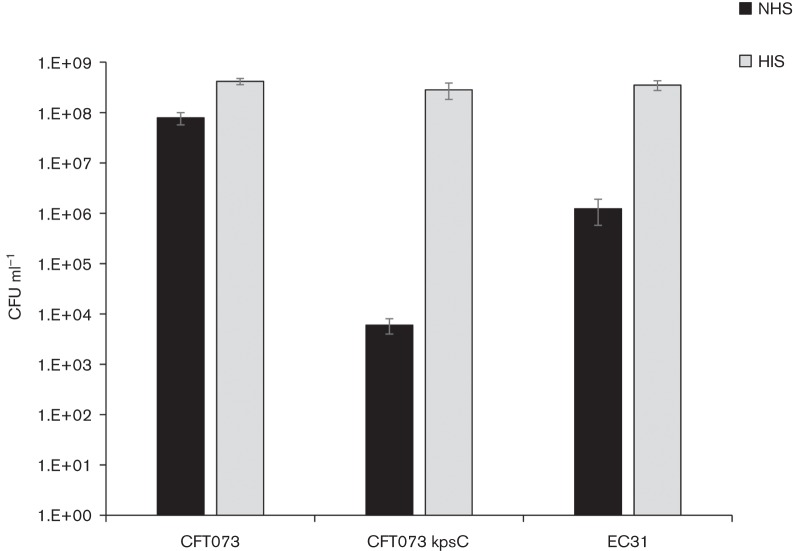
Survival of *E. coli* in the presence of serum. *E. coli* isolates were adjusted to equal concentration in Luria broth and supplemented with 50 % NHS or heat inactivated serum (HIS) (v/v). Samples were incubated at 37 °C for 45 min after which viability was assessed by Miles and Misra method.

Serum resistance of ECBSI31-SJH was also assessed, as resistance is frequently associated with *E. coli* that cause bloodstream infections. Survival in normal human serum (NHS) was compared to survival of serum-resistant and serum-sensitive controls, as described previously (Miajlovic *et al.*, 2014). ECBSI31-SJH was found to be intermediately resistant (Fig. 1).

**Table 1.** Virulence factors detected in ECBSI31-SJH

Category	Virulence factor
Adhesins	Afimbrial adhesin (Afa), Type 1 fimbriae, F9 fimbriae, Iha adhesin, Antigen 43
Iron acquisition	Iron binding protein (SitA), Haem/haemoglobin receptor (ChuA) Yersiniabactin, Aerobactin
Protectins	Colanic acid, Outer membrane protein A (OmpA), Outer membrane protein T (OmpT), Increased serum survival protein (Iss), Conjugal transfer surface exclusion protein (TraT)
Toxins	Secreted autotransporter toxin (Sat), Uropathogen specific protein (Usp)
Other	Pathogenicity island marker (MalX), d-serine deaminase (DsdA)

### Antibiotic resistance in ECBSI31-SJH

Several antibiotic resistance genes were detected in the ECBSI31-SJH genome (Table S2). These included *aac(3)-IId*, *strA*, *strB*, *mph*A, *sul1*, *sul2 *and a novel TEM-family enzyme most closely related to *blaTEM-212* (AHA49909.1). The novel TEM-family β-lactamase is distinguished from TEM-212 by a single Y103S substitution (Fig. S2). The sequence of the ECBSI31-SJH is *blaCTX-M-15*-negative and the strain is susceptible to cefotaxime. While it carries the 58 kb prophage observed in JJ1886 and JJ1887, it lacks the *blaCTX-M-15 *integration seen in these strains (Stoesser *et al.*, 2016). Mutations in *gyrA* and *parC* (GyrA-S83L, D87N; ParC-S80I, E84V) were detected consistent with the fluoroquinolone resistance observed in ECBSI31-SJH.

## Outcome and follow-up

Complete defervescence was seen in the patient following drainage of the cyst, and systemic symptoms resolved within 1–2 days of admission. A further renal ultrasound was carried out 5 days following drain insertion. This ultrasound showed a small residual lesion, with mostly echogenic debris. The drain was removed without complication. Laboratory markers showed peak C-reactive protein of 205 mg l^−1^ within 24 h of admission, which was reduced to 8.26 mg l^−1^ at the time of discharge.

## Discussion

This report outlines the first description of a novel *E. coli* ST-458, which was the causative agent of pyelonephritis and bacteraemia in an 81-year-old man. ExPEC are frequently associated with urinary tract infections (UTIs) and bacteraemia in both the community and healthcare setting. The infection described in this report was considered to be community acquired, since the patient had no known healthcare exposure in the 12 weeks prior to infection.

Antibiotic susceptibility tests indicated that ECBSI31-SJH was an MDR strain. This is not unusual, as high rates of MDR have been reported for *E. coli* that cause bacteraemia (Alhashash *et al.*, 2013). The presence of the *aac(3)-IId* gene in ECBSI31-SJH is consistent with the aminoglycoside resistance phenotype observed while the mutations observed in *gyrA* and *parC* are known fluoroquinolone resistance determinants. The WGS analysis provided additional information regarding the susceptibility of the isolate. The presence of the *strA*, *mph*A, *sul1* and *sul2* genes indicates that ECBSI31-SJH has a broader range of resistance than that screened for by the laboratory Vitek^®^ 2 susceptibility testing system.

ECBSI31-SJH had many of the virulence factors associated with ExPEC that allow it to adhere to host tissues and cells, evade the host immune system, obtain nutrients and cause tissue damage. Amongst the adhesins detected in the WGS analysis, the type 1 fimbriae promote adherence to the urinary tract epithelium and antigen 43 has been implicated in long-term colonisation of the bladder (Mulvey *et al.*, 2000; Ulett *et al.*, 2007). Another indication that ECBSI31-SJH is an uropathogenic strain was the presence of all 131 UPEC-specific genes identified by a previous study (Lloyd *et al.*, 2007). Although the patient had benign prostatic hypertrophy, there was no evidence of a preceding UTI based on urine samples sent from primary care and previous hospital admissions. However, a urine sample taken from the patient following admission was positive for *E. coli* with corresponding antibiotic susceptibility to ECBSI31-SJH. The majority of *E. coli* bloodstream infections originate from the urinary tract and gain access to the bloodstream *via* the bladder and kidneys. The isolation of *E. coli* from a urine sample and from the fluid drained from the kidney cyst suggest a similar progression in this case.

The two toxins identified in ECBSI31-SJH could have contributed to pyelonephritis and tissue damage. The secreted autotransporter toxin (Sat) has a cytopathic effect on kidney cells and contributes to damage to the kidney epithelium in upper UTI (Guyer *et al.*, 2002). The uropathogen-specific protein (Usp) is a genotoxin that has been associated with pyelonephritis, prostatitis and bacteraemia originating in the urinary tract (Nipic *et al.*, 2013).

Amongst the other virulence factors identified in the WGS analysis, there were several factors involved in iron acquisition. Both the urinary tract and bloodstream are iron- limited environments. The presence of factors involved in iron acquisition such as SitA, ChuA, Yersiniabactin and Aerobactin is important in the fitness of *E. coli* in the urinary tract and during systemic infection (Smith *et al.*, 2010; Subashchandrabose *et al.*, 2013). Also important to survival in the bloodstream is the ability to resist the bactericidal action of serum (Johnson, 1991; Miajlovic *et al.*, 2014). ECBSI31-SJH was classed as intermediately serum resistant and had a number of virulence factors that are reported to contribute to serum survival. These included the colonic acid polysaccharide, OmpA, OmpT, Iss and TraT (Miajlovic & Smith, 2014). The majority of *E. coli* isolated from the bloodstream are serum resistant. However, host factors that negatively affect immunity can contribute to the survival of less resistant strains (Miajlovic *et al.*, 2016). In this case, both iron deficiency anaemia and diabetes mellitus could have had a negative impact on the patient’s innate immunity.

In summary, ECBSI31-SJH is the first reported ExPEC of novel ST-458. Though divergent in its MLST profile, ECBSI31-SJH exhibits genomic similarity to ST-131 and retains characteristics of the ST-131 C1/H30R sub-lineage, including multi-drug resistance and the presence of ExPEC virulence factors. These traits are likely to have contributed to the ability of ECBSI31-SJH to gain access to the kidney and bloodstream and cause infection in this case.
